# YOLOv5-FPN: A Robust Framework for Multi-Sized Cell Counting in Fluorescence Images

**DOI:** 10.3390/diagnostics13132280

**Published:** 2023-07-05

**Authors:** Bader Aldughayfiq, Farzeen Ashfaq, N. Z. Jhanjhi, Mamoona Humayun

**Affiliations:** 1 Department of Information Systems, College of Computer and Information Sciences, Jouf University, Sakaka 72388, Saudi Arabia; bmaldughayfiq@ju.edu.sa; 2 School of Computer Science (SCS), Taylor’s University, Subang Jaya 47500, Malaysia; farzeen.ashfaq@sd.taylors.edu.my (F.A.); noorzaman.jhanjhi@taylors.edu.my (N.Z.J.)

**Keywords:** deep learning, automated cell counting, feature pyramid network, YOLOv5, fluorescence images

## Abstract

Cell counting in fluorescence microscopy is an essential task in biomedical research for analyzing cellular dynamics and studying disease progression. Traditional methods for cell counting involve manual counting or threshold-based segmentation, which are time-consuming and prone to human error. Recently, deep learning-based object detection methods have shown promising results in automating cell counting tasks. However, the existing methods mainly focus on segmentation-based techniques that require a large amount of labeled data and extensive computational resources. In this paper, we propose a novel approach to detect and count multiple-size cells in a fluorescence image slide using You Only Look Once version 5 (YOLOv5) with a feature pyramid network (FPN). Our proposed method can efficiently detect multiple cells with different sizes in a single image, eliminating the need for pixel-level segmentation. We show that our method outperforms state-of-the-art segmentation-based approaches in terms of accuracy and computational efficiency. The experimental results on publicly available datasets demonstrate that our proposed approach achieves an average precision of 0.8 and a processing time of 43.9 ms per image. Our approach addresses the research gap in the literature by providing a more efficient and accurate method for cell counting in fluorescence microscopy that requires less computational resources and labeled data.

## 1. Introduction

Cell counting is a fundamental task in biological research and clinical practice. It is an essential step for various applications, such as cell culture [1,2,3], drug discovery [4], disease diagnosis [5,6,7], and treatment monitoring [8,9]. Accurate cell counting is crucial for understanding cellular behavior, identifying cellular abnormalities, and evaluating the efficacy of interventions [10]. Traditionally, cell counting is performed manually by trained personnel using a microscope and a counting chamber, which is time-consuming, labor-intensive, and prone to errors [11]. Therefore, there is a growing need for robust and automated cell counting methods [12,13].

Several traditional methods have been developed for cell counting, such as manual counting [14,15], hemocytometry [16,17], and flow cytometry [18]. Manual counting is the most straightforward method, but it is subject to inter-observer variability, low throughput, and poor reproducibility [19]. Hemocytometry, however, is a more accurate and reliable method, but it is still labor-intensive and requires skilled operators [15,20]. Flow cytometry is a high-throughput and automated method, but it requires expensive equipment and specialized training [21]. Moreover, these methods are often limited by their ability to distinguish different cell types and sizes, especially in complex biological samples.

**Figure 1:**
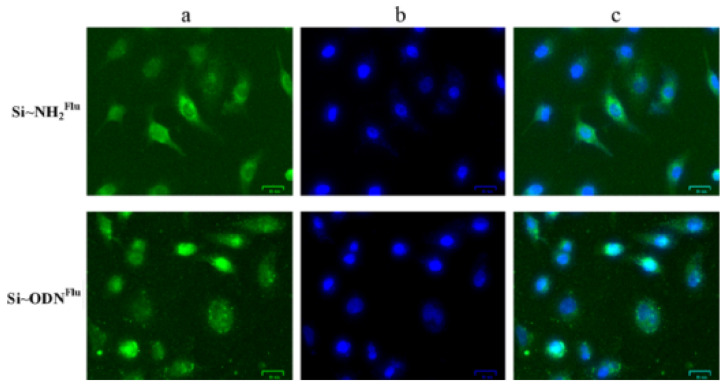
Fluorescence microscopy images of A549 human lung adenocarcinoma cells after their incubation with Si–NH ${}_{2}$ Flu nanoparticles and Si–NH${}_{2}$·ODN(3)Flu nanocomplexes. (**a**) Samples that were fluorescein-labeled were found in the green channel (488 nm). (**b**) The blue channel (405 nm) revealed cell nuclei stained with DAPI. (**c**) All channels superimposed. (Scale bar: 25 m for all). Figure adapted from [24].

Fluorescence cell counting is a more advanced method that uses fluorescent dyes to label specific cell populations and quantify them with the help of fluorescence microscopy [22,23]. This method allows for higher accuracy and specificity, especially in complex biological samples, but it requires specialized equipment and expertise. Figure 1 adapted from [24] shows the fluorescence microscopy image depicting A549 human lung adenocarcinoma cells after their incubation, fluorescence microscopy enables the segmentation of individual cells and the removal of artefacts such as cellular detritus by highlighting the contour of objects based on their fluorescent signal. The picture demonstrates how fluorescence-based cell counting methods can be used to visualise and examine particular cell populations. Fluorescence cell counting has gained popularity as a method in biological research due to the expanding availability of fluorescent dyes and imaging technologies  [25].

Moreover, it can be said that conventional cell counting techniques have a number of drawbacks and difficulties, including poor accuracy, slow throughput, high cost, and subjectivity. Additionally, they might not be appropriate for complex samples, such as tissues, cell aggregates, or mixed cell populations [26]. Therefore, more accurate and automated cell counting methods utilizing a fluorescence mechanism are becoming more and more popular.

More reliable and automated cell counting techniques have emerged as a result of recent developments in computer vision [27] and machine learning [28]. These techniques make use of the capabilities of deep learning algorithms to find and count cells in images from fluorescence microscopy. Deep learning-based approaches for counting are more precise and reproducible than conventional approaches because they can handle a wide range of objects with varying kinds, sizes, and complex materials and textures [29]. When we look at the literature on cell counting using automated tools, the bulk of the methods that are currently being used, however, rely on segmentation-based tactics, which call for a lot of training, tuning, and parameter optimization [30,31,32,33]. These techniques utilize image processing algorithms such as edge detection, thresholding, morphological operations, and watershed segmentation to separate cells from the background and from each other [34,35,36,37,38]. While these techniques have shown promising results, they are often computationally intensive and require skilled personnel for optimization and validation. More advanced methods use machine learning algorithms such as a CNN to accurately identify cells. One such latest algorithm is YOLO (You Only Look Once), which uses a single neural network to detect and classify objects in real time [39,40,41]. Although it has been widely used in object detection and counting tasks [42,43,44,45,46] and  [47], its application in automated cell counting is still being explored, and there is potential for further research in this area. Using a single neural network, the real-time object detection and counting method forecasts the bounding boxes and class probabilities of objects in a picture. It operates by dividing the input image into a grid and forecasting bounding boxes and class probabilities for each grid cell. This can thus make it useful for cell counting, where the objective is to identify and count multiple cells in a given fluorescence image. Moreover, feature pyramid networks have also been employed as an effective method in improving the object detection accuracy of YOLO by leveraging feature maps at different resolutions [48].

In this study, we propose to use YOLOv5 with a feature pyramid network to detect and count multiple-sized cells in fluorescence microscopy images. An FPN is a popular architecture for object detection that utilizes feature maps of different resolutions to improve the accuracy of object detection. We believe that the combination of YOLOv5 and an FPN can enhance the detection and counting of cells of different sizes in a single image.

Our main contributions are highlighted as follows:

- We utilized YOLOv5, a state-of-the-art object detection algorithm, for cell counting in fluorescence microscopy images.
- We employed the FPN as a feature extractor to handle cells of different sizes in the images.
- We annotated the cell images with bounding boxes using a labeling tool for training the YOLOv5 model.
- We augmented the original dataset of 283 images to 600 images with rotation, scaling, and flipping to improve the model’s performance.
- We evaluated the performance of the YOLOv5 model with an FPN on the cell counting task and compared it to other YOLOv5 model versions.

The remainder of this paper is structured in the following manner. Section 2 presents a comprehensive literature review of previous studies on cell counting using traditional methods and deep learning techniques. Section 3 introduces the two main components of our approach, YOLO and the FPN, and explains their technical details. Section 4 outlines our proposed approach in detail, including dataset annotation, data preprocessing, customizing YOLO’s hyperparameters, and the experimental settings. Section 5 presents the results of our experiments and an evaluation of our approach’s performance in terms of accuracy and efficiency. Section 6 discusses the implications of our findings and compares our approach’s performance with existing methods. Finally, Section 7 concludes the paper by summarizing the main contributions, discussing the limitations and future directions, and providing a final remark on the potential applications of our approach in the field of cell counting.

## 2. Literature Review

In many biomedical applications, including cancer detection, drug discovery, and toxicity testing, cell counting is a critical step [45,49,50,51]. Using a microscope and a counting chamber, skilled workers manually count cells according to traditional methods [18]. Although manual cell counting is the industry standard, it is labor-intensive, time-consuming, and prone to human error, making it challenging to standardize and replicate results across various samples [52]. Additionally, it can be difficult to distinguish between cells that are the same size and shape or that group together, which can result in errors in cell counts [53].

Automated cell counting techniques have been developed to address these issues [34,54,55]. One of the earlier techniques was based on electronic particle counting, which detects cells as they pass through a small aperture using impedance or light scattering. Although this method is quick and precise, it cannot tell the difference between live and dead cells and needs a high cell density [56].

**Figure 2:**
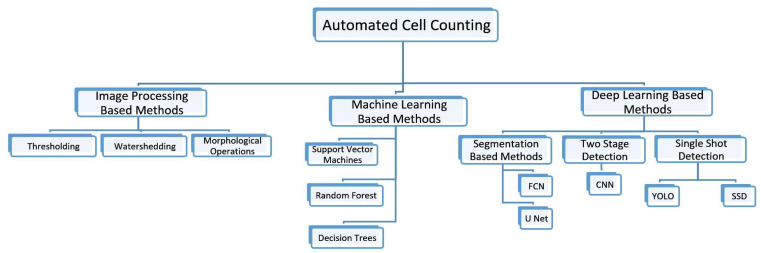
Classification of Methods of Automated Cell Counting.

The examination of digital photographs of cells using computer algorithms is a different automated way for counting the number of cells [57]. Direct and indirect procedures make up the two basic groups into which these techniques can be divided. With direct methods, cells are marked with stains or dyes and counted in accordance with their fluorescence or absorbance. The investigation of morphological characteristics of cells, such as their size, shape, and texture, is utilized in indirect approaches for cell counting and can be used to recognize and count cells in digital images. Deep learning, machine learning, and image processing-based methods are another way to categorize automated cell counting techniques. The classification tree is shown in Figure 2.

Image processing based methods involve the application of mathematical operations to enhance, segment, and analyze cell images [58,59]. These methods are computationally efficient but require expert knowledge to design and tune the algorithms [60]. Machine learning based methods use statistical models to learn patterns and features from the data to classify and count cells. These methods require extensive feature engineering and parameter tuning, making them time-consuming and computationally expensive [61,62]. On the other hand, deep learning-based methods use artificial neural networks with multiple layers to automatically learn and extract features from the data. These methods have shown superior performance in terms of accuracy and speed compared to other methods. They have shown remarkable results in various other domains also, particularly in medical image analysis [63,64,65]. Table 1 summarizes the performance of the three methods in terms of advantages and limitations and also highlights their applications in cell detection and counting.

Morelli et al. in [70] suggest using deep learning to automate cell counting in fluorescence microscopy. To localize cells and obtain counts as the number of observed objects, the method employs a fully convolutional network known as c-ResUnet. Kayasandik et al. in [72] provide a unique image analysis framework for automatic astrocyte recognition and segmentation in 2D fluorescent brain tissue images. The method contains two significant innovations: an automated cell detection method based on multiscale directional filters and astrocyte segmentation using a modified CNN architecture. In another work by [73], a deep learning-based approach is proposed for creating pseudo-nuclear stained images from phase contrast images of cells. To recognize the nuclei of cells, the suggested method employs a simple deep neural network. The suggested approach also determines the relative position of the cells, counting the number and tracking them for different cell densities. Using fluorescence microscopy pictures, Ref. [74] demonstrates an automated workflow for recognizing and counting Mycobacterium tuberculosis (Mtb) germs in sputum samples. The pipeline is divided into four stages: annotation with generative adversarial networks (GANs), extraction of key picture patches, classification of extracted patches, and regression to obtain the final bacteria count. In a very similar work, Ref. [75] proposes a framework that uses a DCNN for automated cell counting in time-lapse microscopy images of developing human embryos. The study uses a dataset of 265 human embryos to demonstrate the effectiveness of the approach. The results show that the proposed framework provides robust estimates of the number of cells in a developing embryo up to the 5-cell stage, which is 48 hours post-fertilization. Whereas, Ref. [76] uses a fully convolutional regression network to estimate cell density maps from images. The method includes auxiliary convolutional neural networks to improve performance on unseen datasets.

Furthermore, Refs. [71,77] propose an automated method for blood cell counting and categorization that employs instance segmentation, transfer learning, and Mask R-CNN. The proposed approach successfully detects a wide range of blood cells, including overlapped and faded cells. Similarly, Ref. [78] establishes a 3D cell counting method based on U-net deep learning to effectively identify original seed cell numbers in extracellular matrix (ECM) aggregated cells. When compared to standard contour and watershed segmentation methods, the proposed method has a smaller counting error.

Finally, Ref. [79] presents a deep learning approach for the detection and segmentation of macrophage cells in fluorescence microscopy images using feature pyramid fusion. The proposed approach shows superior performance compared to a state-of-the-art Mask R-CNN approach and provides a novel dataset of macrophage cells for public use. Likewise, Ref. [80] proposes a fully convolutional neural network-based approach for automatic cell counting in fluorescent microscopy images. The proposed method shows human-level performance and satisfactory performance in terms of the counting task, with mean and median absolute errors of 0.8 and 1, respectively.

Despite all the preceding literature, the task of cell counting in fluorescence images has yet to be studied utilizing a single-stage method for object detection. One such object detection technology is You Only Look Once (YOLO), a cutting-edge deep learning-based object identification framework. Because it uses a single convolutional neural network to estimate bounding boxes and class probabilities directly from complete pictures, YOLO is faster and more accurate than earlier object detection algorithms [40]. Numerous applications, including self-driving cars, pedestrian detection, and face detection, have effectively exploited YOLO for object detection [81,82,83].

FPNs are another deep learning-based approach for image object detection, segmentation, and feature extraction. An FPN is a multiscale pyramid network that detects objects of varying sizes in images by using feature maps of varying resolutions [84]. An FPN has been used to recognize and segment small objects in aerial photos and to segment buildings in satellite images [85,86,87].

## 3. Baseline Architecture

**Figure 3:**
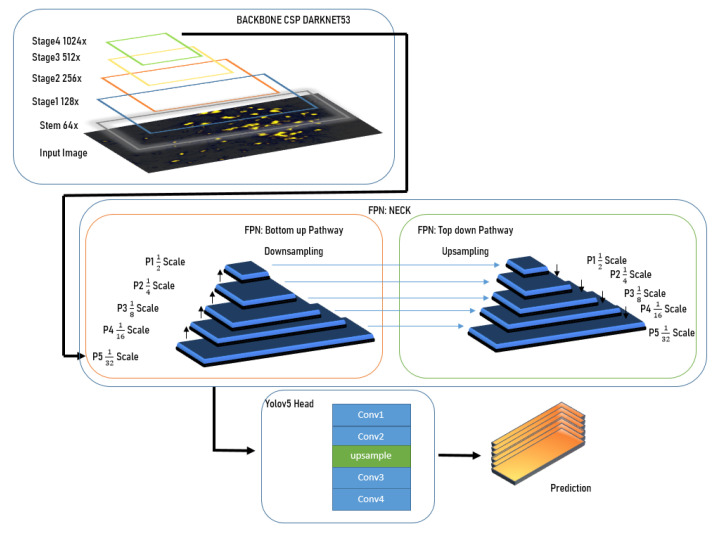
Architecture of our YOLOv5 model with FPN.

Figure 3 illustrates the baseline architecture of our model.

### 3.1. Overview of YOLOv5 Architecture

The architecture of YOLOv5 follows a similar concept to previous versions of YOLO, where a single neural network is trained to directly predict bounding boxes and class probabilities for each object in the image. However, YOLOv5 has undergone several improvements to make it faster and more accurate. The network architecture of YOLOv5 is based on a backbone of CSP convolutional layers and a neck of PAN layers, followed by three different-sized detection heads. This architecture allows the model to capture features at different scales, leading to improved accuracy in object detection tasks.

### 3.2. Overview of FPN Architecture and Implementation

The basic idea behind an FPN is to construct a pyramid of multiscale feature maps from a single input image. This pyramid is built by applying a series of convolutional layers with decreasing spatial resolution to the input image. The resulting feature maps at each level of the pyramid contain different levels of semantic information and different levels of detail. The top-level feature map has the lowest spatial resolution but the highest semantic information, while the bottom-level feature map has the highest spatial resolution but the lowest semantic information.

### 3.3. Combining YOLOv5 and FPN

YOLOv5 with an FPN and YOLOv5 head architecture consists of three main parts: backbone, neck, and head.

#### 3.3.1. Backbone

The backbone is responsible for extracting feature maps from the input image. YOLOv5 uses a modified CSP backbone that is composed of a series of convolutional layers with shortcut connections. The backbone consists of four stages, where each stage performs downsampling of the feature maps. The stem is the initial part of the backbone that processes the input image and generates the first set of feature maps. In YOLOv5, the stem consists of a series of convolutional layers and a pooling layer that downsamples the image to a smaller size. The stem is responsible for extracting low-level features such as edges and corners from the input image. The stages are responsible for progressively extracting more complex features from the input image by processing the feature maps generated by the previous stage. Each stage typically performs downsampling of the feature maps to increase their receptive field and reduce their spatial resolution. The stem and stages in the YOLOv5 backbone are responsible for extracting increasingly complex features from the input image, which are then used by the feature pyramid network (FPN) and YOLOv5 head to generate bounding box predictions and class probabilities.

#### 3.3.2. Neck

The neck connects the backbone to the head and is responsible for fusing feature maps of different resolutions. We use a feature pyramid network (FPN) as the neck, which generates a pyramid of feature maps at different scales by combining feature maps from different levels of the backbone. The FPN consists of two parts: a bottom-up pathway that generates the feature maps from the backbone, and a top-down pathway that combines the feature maps to create a pyramid of features. After the last stage of the backbone, the feature map has a very small spatial resolution, which makes it difficult to detect small objects. To address this, the FPN upsamples the feature maps from the lower scales and fuses them with the feature maps from the higher scales to create a set of feature maps with varying scales and resolutions. In the FPN, the upsampling operation is used to increase the spatial resolution of the feature maps from the lower scales to match the resolution of the feature maps from the higher scales. The upsampled feature maps are then merged with the higher-scale feature maps using an additional operation to create a fused feature map.

The merged feature map is then passed through a convolution layer to refine the features and reduce the channel depth to match the desired output size. The resulting feature map is then used as the input to the next level of the pyramid.

The downsampling operation, which reduces the spatial resolution of the feature maps, is typically implemented using pooling layers, such as max pooling or average pooling. This operation is used in the backbone to reduce the spatial resolution of the input image and generate the initial feature maps.

#### 3.3.3. Head

The head is responsible for predicting the bounding boxes and class probabilities for the objects in the input image. The YOLOv5 head architecture is a single-stage object detection system that predicts bounding boxes and class probabilities directly from the fused feature maps generated by the FPN. The head consists of a series of convolutional layers that reduce the dimensionality of the fused feature maps, followed by two fully connected layers that output the bounding box coordinates and class probabilities.

## 4. Methodology

### 4.1. Dataset Preparation

The dataset utilized for cell counting in this study was obtained from the study conducted by [70], consisting of 283 images of cultured cells from mice brain slices. As the dataset was not initially suitable for object detection tasks using bounding boxes, we preprocessed the data by annotating individual cells using the LabelImg tool in YOLOv5 pytorch format. This involved converting the bounding box coordinates to the format (x,y,w,h), where (x,y) represents the center coordinates of the bounding box and (w,h) represents the width and height of the bounding box relative to the image size. The class label for each bounding box was also encoded as an integer. A thorough quality check was conducted to ensure the accuracy and consistency of the annotations. Following annotation, we randomly divided the dataset into training (80%), validation (10%), and testing (10%) sets in order to train and evaluate our deep learning model for autonomous cell counting.

### 4.2. Dataset Augmentation

In order to increase the diversity of the dataset and prevent overfitting, data augmentation techniques were applied to the original dataset of 283 images. The following data augmentation techniques were used:

1. Horizontal flipping: The images were horizontally flipped to generate new images.
2. Rotation: The images were rotated at different angles to create variations in the cell positions and orientations.
3. Brightness and contrast adjustment: The brightness and contrast of the images were adjusted within a range of −40 to +40 to simulate different lighting conditions and highlight the dim and dull cells.

The augmented dataset was generated by applying these techniques randomly to the original dataset. The size of the augmented dataset was increased from 283 to 600 images. The effectiveness of the data augmentation techniques was evaluated by training the custom YOLOv5 model on both the original and augmented datasets and comparing their performance.

### 4.3. Customizing YOLOv5

To customize YOLOv5 for cell counting, we needed to modify the architecture and parameters of the model. The YOLOv5 architecture consists of a backbone network and a detection head. The backbone network is responsible for feature extraction from the input image, while the detection head is responsible for predicting the bounding boxes and confidence scores for objects in the image. We customized both parts of the network to improve its accuracy for cell counting. To configure YOLOv5 for cell counting, we also modified the number of classes in the detection head to one, as we are only interested in detecting one type of object, i.e., cells. We also modified the anchor box sizes and aspect ratios to better match the size and shape of cells in our images. In addition, we modified the output layer of the network to predict the number of cells in the image instead of detecting their bounding boxes. Configuring the YOLOv5 architecture with FPN for cell counting, the baseline architecture was modified to include the FPN module for multiscale feature extraction. The number of feature levels and the feature map sizes for each level were determined based on the input image size and the cell size. The algorithm for the modification is presented in Algorithm 1.
**Algorithm 1** Customized YOLOv5 Model with FPN.1:Load the pre-trained YOLOv5 model and remove the detection head.2:Add the FPN layers to the model architecture by implementing the bottom-up and top-down pathways.3:Concatenate the feature maps generated by the bottom-up pathway with the corresponding feature maps generated by the top-down pathway.4:Apply convolutional layers to the concatenated feature maps to generate the final multiscale feature maps.5:Add the detection head back to the model architecture and train the model on the annotated and augmented dataset.6:Evaluate the performance of the customized YOLOv5 model with FPN.

## 5. Results

**Figure 4:**
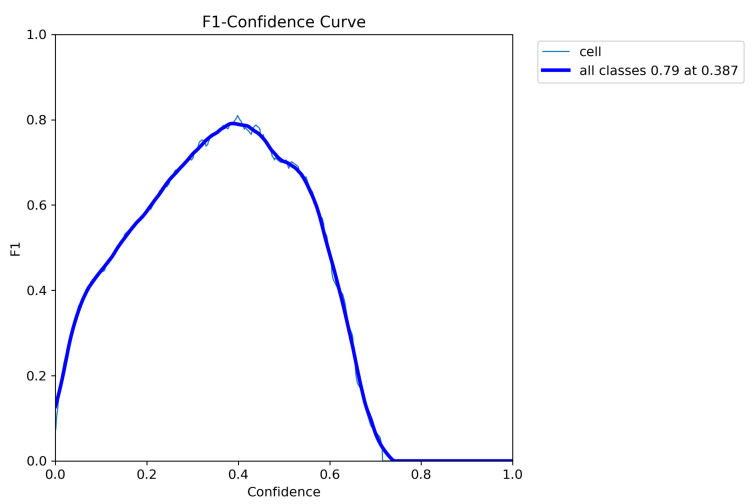
F1 curve illustrating the model’s performance in terms of the F1 score at different confidence thresholds. The F1 curve represents the harmonic mean of precision and recall, providing a balanced measure of the model’s accuracy. Higher values on the curve indicate better overall performance. At a confidence threshold of 38.7%, the model achieves an F1 score of 79%, indicating a good balance between precision and recall.

**Figure 5:**
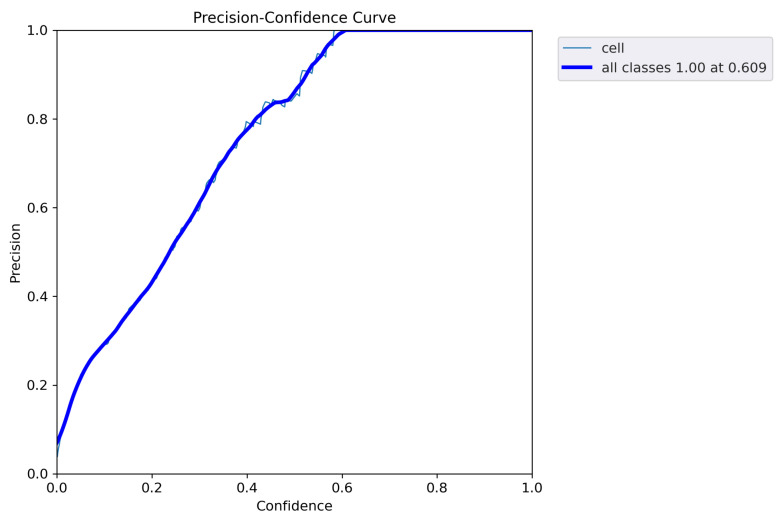
Precision curve illustrating the model’s performance in terms of precision at different confidence thresholds. The precision curve represents the precision values obtained by the model at various confidence levels. Higher values on the curve indicate better precision performance. At a confidence threshold of 60.9%, the model achieves a precision of 100%, indicating perfect accuracy in its positive predictions.

**Figure 6:**
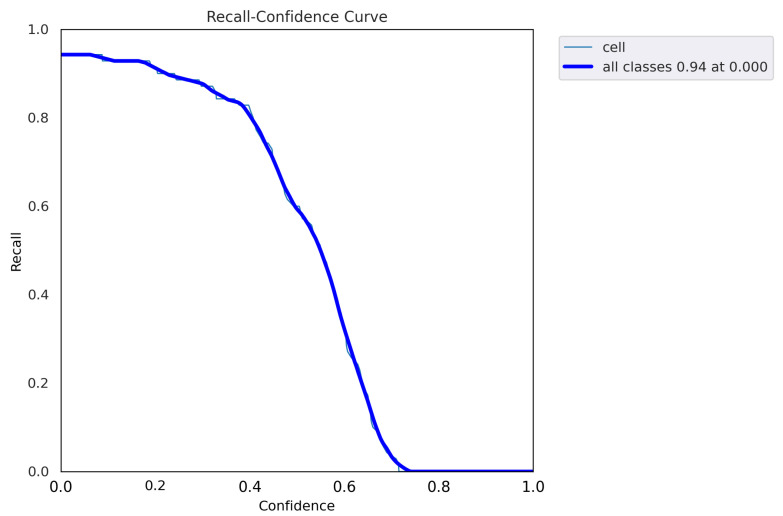
Recall curve illustrating the model’s performance in terms of recall at different confidence thresholds. The recall curve measures the ability of the model to correctly identify positive instances (cells) at varying confidence levels. Higher values on the curve indicate better recall performance. The model achieves a recall of 94%, reflecting its high accuracy in identifying positive instances.

**Figure 7:**
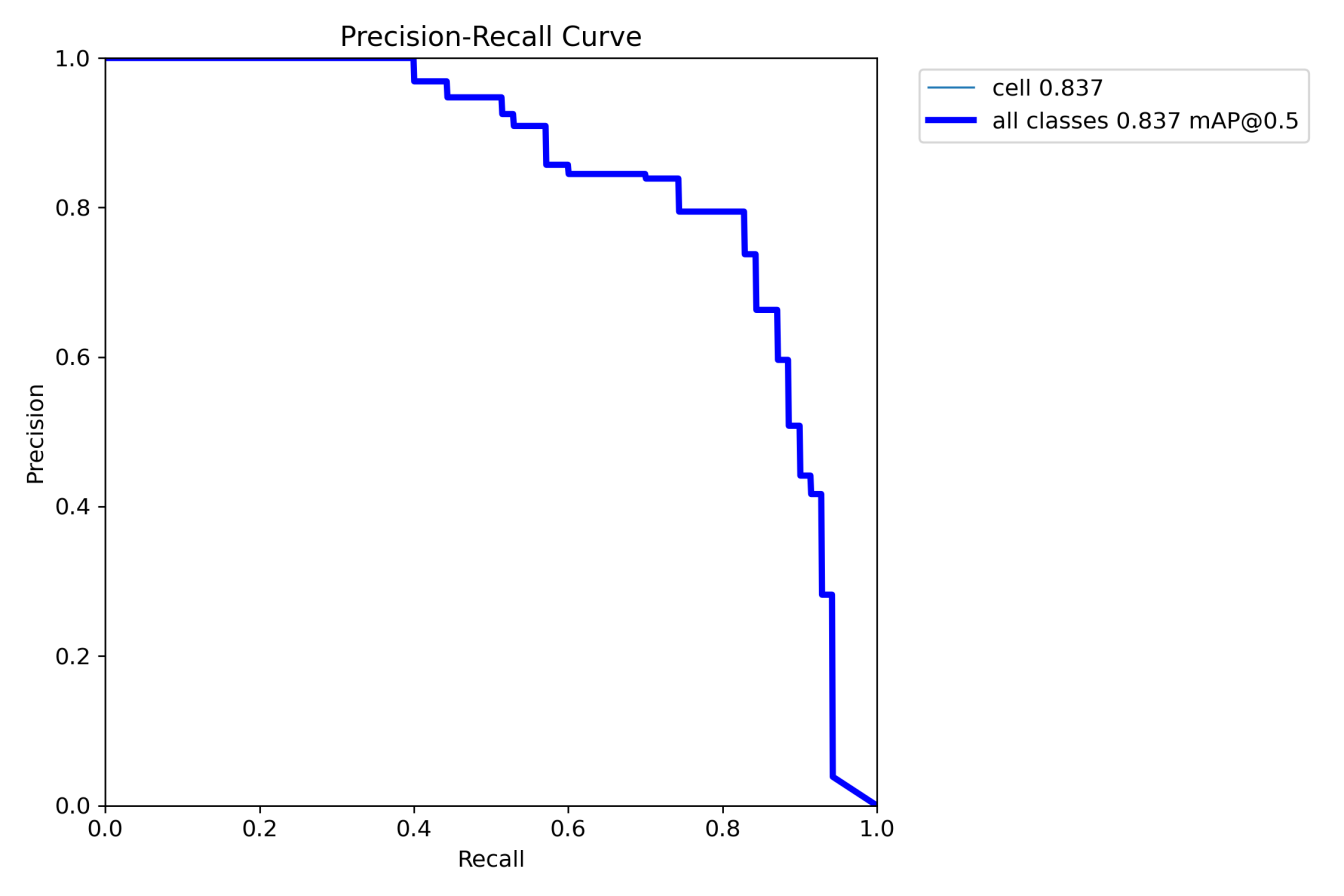
Precision–recall curve illustrating the performance of the proposed model in cell detection. The curve showcases the trade-off between precision and recall, with higher values indicating better performance. At the classification threshold of 0.5, the model achieves a precision of 83.7% and a corresponding recall value.

**Figure 8:**
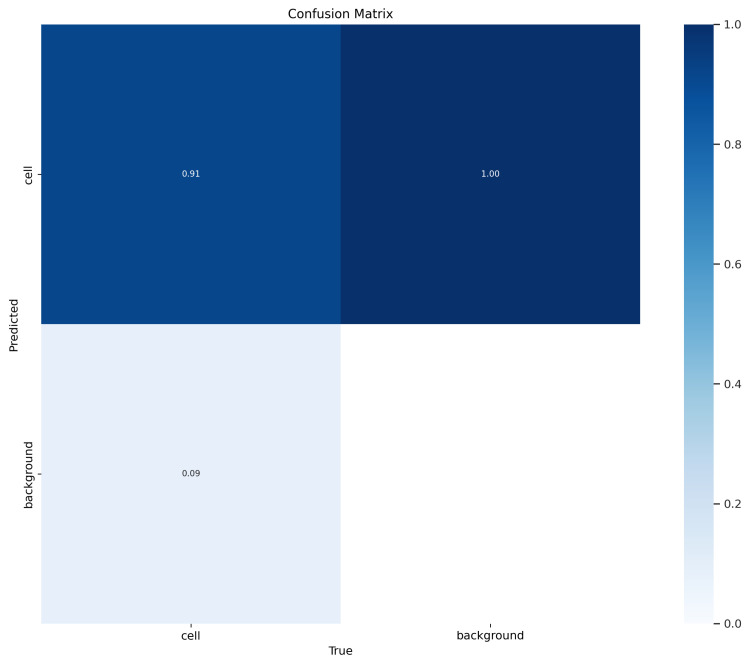
Confusion matrix evaluating the proposed model’s performance. Rows represent actual classes, and columns represent predicted classes. Values in the matrix indicate the number of instances per class. Higher diagonal values indicate accurate predictions, while off-diagonal elements represent misclassifications. Notably, the model achieved a 91% correct detection rate for cell detection.

After fine-tuning the hyperparameters and experimenting with various optimization algorithms, we settled on using the Adam optimizer with an initial learning rate of 0.001, a weight decay of 0.0003, and a batch size of 80 for our customized YOLOv5 model with an FPN. We trained the model on images of different sizes, namely 415 × 415, 640 × 640, and 840 × 840, to generate multiscale feature maps that can detect cells of varying sizes. Figure 4, Figure 5, Figure 6, Figure 7 and Figure 8 illustrate the results obtained from the evaluation of the model on the validation and test datasets. In the validation dataset, the precision was 0.796, recall was 0.741, and mAP was 0.79. In the test dataset, the precision was 0.79, recall was 0.829, and mean average precision was 0.837. The experiments in this study were conducted on a Google Colab platform, utilizing a free GPU Tesla T4 for evaluating the performance of the proposed model. On average, the processing time for each image, encompassing detection and counting, was measured to be 43.9 ms. The confusion matrix in Figure 8 shows that the model correctly predicted the “Cell” class 0.91 times.

The F1 curve illustrates the trade-off between precision and recall for different confidence thresholds. The highest F1 score achieved was 80%, indicating a good balance between precision and recall. The training and validation accuracy and loss curves show the progress of the model’s performance during training. The validation accuracy steadily increased over time, while the training loss steadily decreased, indicating that the model was effectively learning from the data. The precision and confidence curves show the relationship between precision and confidence for different confidence thresholds. The highest precision was achieved at a confidence threshold of 0.7, indicating that the model was highly confident in its predictions at this threshold.

Furthermore, we retrained the model using the initial weights and conducted training for 300 epochs, implementing the early stopping technique. Early stopping involves monitoring the validation loss, and if there is no improvement in the validation loss over a consecutive number of epochs, training is halted. This approach ensures that the model achieves the best performance while preventing overfitting. We also compared the performance of our model with and without data augmentation, and with different image sizes and YOLOv5 architectures (Yolos, Yolon, Yolol, and Yolofpn). Our experiments demonstrated that YOLOv5 with an FPN achieves the best performance with an mAP of 0.799. The comparison table of the results obtained from the different experiments provides a comprehensive view of the performance achieved by our model. Table 2 shows that the model trained with data augmentation achieved higher precision, recall, and F1 scores compared to the model trained without data augmentation. It also shows that the best results were achieved with the YOLOv5 architecture and an image size of 416 × 416, which achieved the highest mAP on the validation set and test set also.

**Figure 9:**
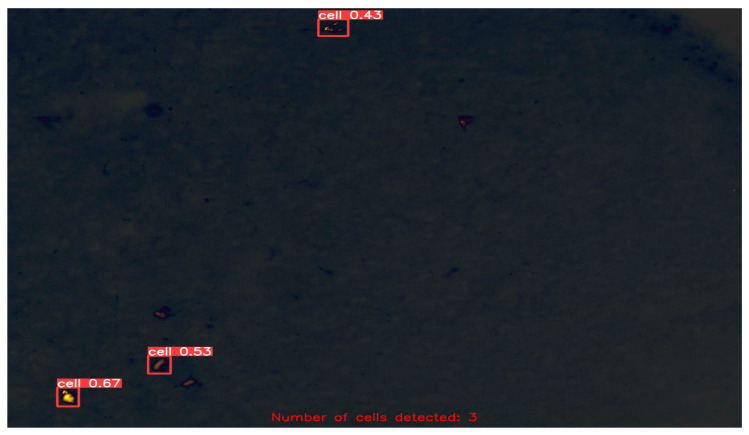
Detected and Counted Cells in Sample Images from Test Dataset = 3.

**Figure 10:**
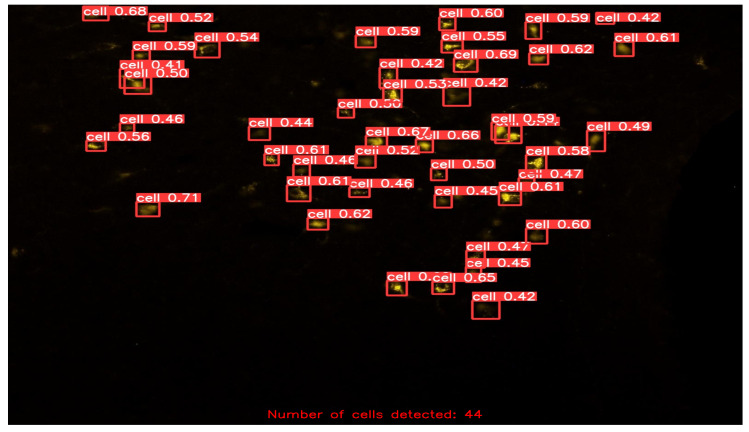
Detected and Counted Cells in Sample Images from Test Dataset = 44.

**Figure 11:**
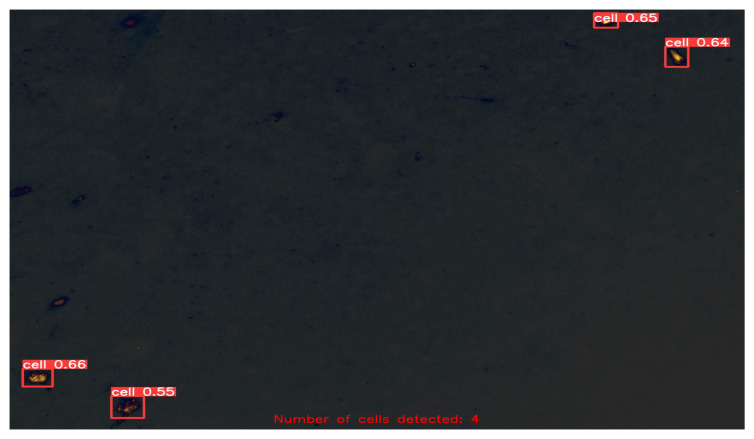
Detected and Counted Cells in Sample Images from Test Dataset = 4.

**Figure 12:**
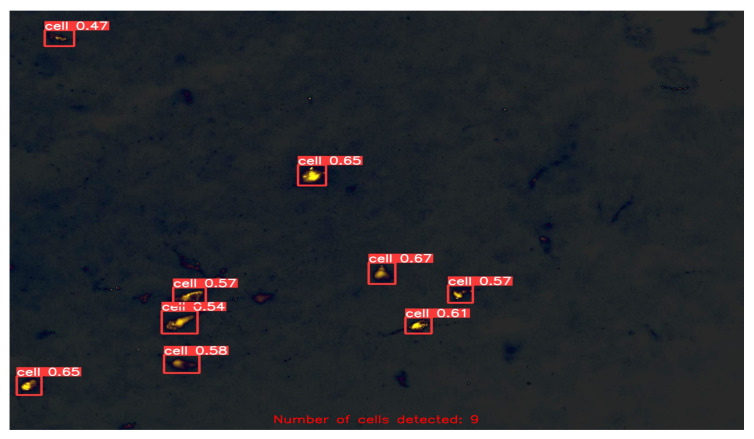
Detected and Counted Cells in Sample Images from Test Dataset = 9.

In the final stage of our evaluation, we quantified the number of cells detected in the test dataset. A few exemplary images from the test dataset are shown in Figure 9, Figure 10, Figure 11 and Figure 12, along with their corresponding detected cells. The numbers with every box along with the word “cell” represent the confidence scores associated with each cell detection. The term “confidence score” in object detection refers to a numerical number that expresses the algorithm’s opinion regarding the likelihood that a detected region or bounding box contains a certain object of interest, in this case “cell”. This analysis allowed us to assess the effectiveness of our customized YOLOv5 model with an FPN in detecting cells accurately and reliably, and to further confirm its potential for use in practical applications.

## 6. Discussion

Our paper presents a machine learning approach for automated cell counting in fluorescent microscopy images using YOLOv5fpn. In comparison to the approach proposed in the UNet paper [80], which achieves an F1 score of 0.87, our method achieves a true positive cell prediction rate of 0.94, demonstrating high precision and accuracy. Our method employs a single-shot detection approach, which eliminates the need for a separate segmentation step and makes the model faster and more efficient. Furthermore, we conducted several experiments to optimize our model’s performance, including retraining the model with early stopping and comparing the performance with and without data augmentation, and with different image sizes and YOLOv5 architectures. Our method employs a single-shot detection approach, which eliminates the need for a separate segmentation step and makes the model faster and more efficient. By directly detecting cells in a single pass, our approach significantly reduces the computational complexity and processing time, making it suitable for large-scale analyses and real-time applications.

Another notable advantage of our approach is its ability to handle cells of varying sizes. Evident from Figure 9, Figure 10, Figure 11 and Figure 12, the cells in the images exhibit size variations and our method successfully detects and counts them. This capability is crucial in biomedical research, as it enables the analysis of cellular populations with diverse sizes and facilitates the study of cellular dynamics and disease progression. Moreover, our method can extract the bounding box coordinates for each detected cell. These coordinates provide the spatial location of the cells within the image, enabling further spatial analysis and characterization. Researchers can use these coordinates to study the distribution and clustering patterns of cells in the image, which can provide valuable insights into cellular dynamics and interactions. Additionally, by measuring the width and height of the bounding box, researchers can obtain an approximation of the size of each cell. This information can be used to analyze cell size distribution, track changes in cell size over time, or compare the sizes of cells under different experimental conditions.

As a whole, our suggested method detects and counts cells accurately while also providing valuable parameters, such as bounding box coordinates, confidence ratings, and projected cell sizes. These parameters enable the comprehensive analysis and characterization of cellular features, facilitating further investigations into cellular dynamics, disease progression, and the effects of various treatments or interventions. In summary, our single-shot detection method shows promise for automated cell counting in fluorescence microscopy pictures, offering a quick and accurate solution that might be employed in a range of research and clinical situations.

## 7. Conclusions

In conclusion, our study presents a promising solution for automated cell counting in fluorescence microscopy images using the YOLOv5fpn model. Our experiments demonstrate the effectiveness of the single-shot detection approach, which eliminates the need for a separate segmentation step, making the model faster and more efficient. We also show that the model’s performance can be further optimized by retraining with early stopping and utilizing data augmentation, achieving good results.

However, our study also has limitations that can be addressed in future work. One limitation is the size and variety of the dataset used for training and validation. While our dataset includes a large number of images, it is limited to a specific type of cell, and future studies could benefit from incorporating additional cell types and imaging conditions. Additionally, our study focuses on detecting and counting cells in 2D images, and future work could explore extending this approach to 3D images or time-lapse microscopy. Overall, our study provides a foundation for further research in the automated analysis of fluorescence microscopy images, with potential applications in various research and clinical settings.

## Figures and Tables

**Table 1:** Pros and Cons of Automated Cell Detection and Counting Methods.

Methods	Advantages	Limitations	Applied to Cell Detection and Counting
Image Processing based	Simple and computationally efficient	Limited accuracy and robustness	[54,66,67]
	No need for large datasets or complex algorithms	Struggle with complex cell morphologies and low SNR	
	Easy to implement and interpret		
Machine Learning based	Can handle complex cell morphologies and low SNR	Requires labeled training data	[52,61,68]
	More accurate and robust than image processing based	Sensitive to variability in data and imaging protocol	
	Can be adapted to different imaging modalities	Requires feature engineering, which can be time-consuming	
Deep Learning based	State-of-the-art accuracy for cell detection and counting	Highly dependent on the quality and quantity of training data	[69,70,71]
	Highly robust to variability in data and imaging protocol	Can be computationally expensive	
	Does not require feature engineering, saving time and effort	May be less interpretable than traditional methods	

**Table 2:** Comparison of performance metrics (precision, recall, mAP) for YOLOv5s, YOLOv5n, and YOLOv5fpn on different input image sizes (416 × 416, 640 × 640, and 840 × 840).

	416 × 416			640 × 640			840 × 840		
	**Precision**	**Recall**	**mAP**	**Precision**	**Recall**	**mAP**	**Precision**	**Recall**	**mAP**
YOLOv5s	0.741	0.701	0.732	0.787	0.744	0.764	0.756	0.723	0.741
YOLOv5n	0.738	0.661	0.681	0.779	0.695	0.73	0.759	0.734	0.749
YOLOv5fpn	0.796	0.741	0.799	0.758	0.740	0.748	0.748	0.708	0.732

## Data Availability

Not applicable.

## Abbreviations

The following abbreviations are used in this manuscript:

2D	Two Dimensional
3D	Three Dimensional
CNN	Convolution Neural Network
CSP	Cross-Stage Partial
DCNN	Deep Convolution Neural Network
FCN	Fully Convolution Network
FPN	Feature Pyramid Network
GAN	Generative Adversarial Network
IoU	Intersection Over Union
PAN	Path Aggregation Network
mAP	Mean Average Precision
Mtb	Mycobacterium tuberculosis
SSD	Single-Shot Detector
UNET	U-Shaped Convolutional Network
YOLO	You Only Look Once
